# Expression signatures and roles of microRNAs in inflammatory breast cancer

**DOI:** 10.1186/s12935-018-0709-6

**Published:** 2019-01-31

**Authors:** Yihang Qi, Xiangyu Wang, Xiangyi Kong, Jie Zhai, Yi Fang, Xiaoxiang Guan, Jing Wang

**Affiliations:** 1 Department of Breast Surgical Oncology, National Cancer Center/National Clinical Research Center for Cancer/Cancer Hospital, Chinese Academy of Medical Sciences and Peking Union Medical College, Beijing, 100021 China; 2 Department of Laboratory Medicine, Mayo Clinic, Rochester, MN 55902 USA; 3 Department of Oncology, The First Affiliated Hospital of Nanjing Medical University, Nanjing, 210029 China

**Keywords:** Inflammatory breast cancer, miRNA/microRNA, Expression, Prognosis, Diagnosis

## Abstract

Inflammatory breast cancer (IBC) is an infrequent but aggressive manifestation of breast cancer, which accounts for 2–4% of all breast cancer cases but responsible for 7–10% of breast cancer-related deaths, and with a 20–30% 10-year overall survival compared with 80% for patients with non-IBC with an unordinary phenotype, whose molecular mechanisms are still largely unknown to date. Discovering and identifying novel bio-markers responsible for diagnosis and therapeutic targets is a pressing need. MicroRNAs are a class of small non-coding RNAs that are capable to post-transcriptionally regulate gene expression of genes by targeting mRNAs, exerting vital and tremendous affects in numerous malignancy-related biological processes, including cell apoptosis, metabolism, proliferation and differentiation. In this study, we review present and high-quality evidences regarding the potential applications of inflammatory breast cancer associated microRNAs for diagnosis and prognosis of this lethal disease.

## Introduction

Breast cancer, a malignant breast neoplasm originating from breast tissues, is the cause of cancer-related mortality among women worldwide. It’s also a highly heterogeneous disease that consists of multiple subtypes with distinguishing clinical effectiveness and distinct prognosis. On the basis of the American joint committee on cancer (AJCC), inflammatory breast carcinoma (IBC) is “a clinic pathologic entity characterized by diffuse erythema and edema of the breast, often without an underlying palpable mass”, which is a rare but lethal form of primary breast neoplasm, which only accounts for 2–4% of all breast cancer cases but responsible for 7–10% of breast cancer-related deaths [1]. The median overall survival of IBC patients is approximately 2.9y compared with 6.4y for patients with locally advanced breast cancer (LABC) [2], but the molecular mechanisms are still largely unknown to date [3, 4]. Even with a great deal improved therapies, including surgery, radiation, hormone therapy, anthracycline-based or herceptin-based chemotherapy and combination of these modalities, IBC patients still have relatively poor survival outcomes, which is related to its biological characters, such as lymph angiogenesis, intense angiogenesis and vasculogenesis [5–9].

IBC is known as a heterogeneous disease histomorphologically, which is also manifested in the molecular level. Some published studies about mRNA expression profiling to date have showed that transcript-heterogeneity exists in IBC as comprehensively as in non-IBC. Apart from that, the molecular subtypes established such as HER2-positive, luminal, and basal type can also be identified in IBC [10–20]. Furthermore, several previous studies have demonstrated a particular expression signature of miRNAs in IBC and non-IBC, when compared to healthy controls as well as among this two forms of breast cancer. Compared with healthy controls, serum levels of some miRNAs were obvious down-regulated in IBC, while some miRNAs were apparent over-expressed, which indicates that these miRNAs may probably be used as a new bio-marker for diagnosis or prognosis of IBC and non-IBC [21].

MicroRNAs (miRNAs) are single-stranded small and non-coding RNAs that are responsible for regulating the gene expression post-transcriptionally. They exert their regulatory functions via sequence-specific interactions with congenetic target mRNAs, usually by binding to the 3′untranslated regions of the target mRNAs [22], thus stimulating target mRNAs translational repression and degradation [23]. Aberrances of miRNAs expression in tumor versus normal breast tissues are in connection with the occurrence and development of neoplasm [24] and closely related to invasiveness [25], molecular subtypes [26] and hormone receptor status of breast cancer [27, 28]. Furthermore, certain miRNAs also function as tumour oncogenes or suppressors in different cancers. For example, microRNA-133a acts as a tumour suppressor in breast cancer through targeting LASP1 [29]. All these pathological events due to a wide array of pathological and biological processes such as apoptosis, cell proliferations and tumorigeneses resulted from dysregulated miRNA expression [30], accounting for the possibility of miRNAs serving as valued molecular bio-markers for diagnosis and prognosis of tumors.

Increasing evidence has been shown that miRNAs hold great promise to serve as distinctive and non-invasive molecular bio-markers for tumor [31, 32], which is mainly based on the following advantages. (1) The sequences of lots of miRNAs are conserved across different species, which means subtle individual differences in the population; (2) the expression of some miRNAs is related to many diseases closely, especially in tumors, and the expression is also restricted to specific tissues or biological stages of ailments. (3) Originated from cancer tissue [33], miRNAs are stably existed in serum/plasma because of their strong resistance to RNase digestion as well as their protection from degradation by means of inclusion in various protein complexes or numerous membranous particles such as micro vesicles or exosomes. (4) Serum/plasma samples are available conveniently and non-invasively, and the levels of miRNAs can also be easily measured by multifarious common and mature detection technology [34, 35]. All these findings show that, as a novel, safe and non-invasive bio-marker, miRNAs will furnish considerable diagnostic and prognostic values on IBC and non-IBC diseases by restoring or antagonizing their expression [36, 37].

## miRNAs associated with the diagnosis of IBC

Only several researchers have identified the importance of miRNAs come from IBC tissue as diagnostic molecular bio-markers so far. Thus, evidence of evaluating the diagnostic effect of miRNAs in blood, other bio-fluids and primary tissues from IBC patients is considerably devoid. Given the fact that the diagnostic potential of IBC miRNAs is undiscovered and underestimated at present, it is of vital importance to perform substantial studies to identify miRNA-based bio-markers available for early diagnosis of IBC. In limited circumstances, here we concluded 5 miRNAs that is capable of being diagnostic molecular bio-marker for IBC patients which are confirmed by several previous high-quality studies (Table 1; Fig. 9).

**Table 1:** Compendium of miRNAs with potential as bio-markers for inflammatory breast cancer

miRNAs	Breast cancer type	Sample	Roles	Expression	Expression signatures and clinical correlations	Predicted targeted genes	Pathways	References
miR-301b	IBC/non-IBC	Primary tissue	Diagnosis	Up-regulated	Highly over-expressed in IBC samples, high expression was associated with higher grade in IBC, and higher grade, ER negativity and stage III in non-IBC	FOXF2, BBC3, PTEN, COL2A1	Proliferation, invasion	[39, 40]
miR-451	IBC	Serum	Diagnosis	Down-regulated	The most down-regulated miRNAs in IBC, able to enhance tamoxifen sensitive	MIF, YWHAZ	Proliferation, colony formation, invasion	[21, 37, 41, 42]
miR-15a	IBC	Serum	Diagnosis	Down-regulated	Lower in HER2+ IBC patients	CCNE1, CDCA4, BCL2L2, YAP1, AKT-3, SNCG	Proliferation, invasion, apoptosis	[37, 43–46]
miR-342-3p	IBC	Plasma	Diagnosis	Down-regulated	Lower in pre-menopausal IBC patients, associated with ER levels	ID4	Apoptosis	[37, 47]
miR-342-5p	IBC/non-IBC	Serum	Diagnosis	Down-regulated	Lower in post-menopausal IBC patients, associated with ER levels	ID4	Apoptosis	[37, 47]
miR-24	IBC/non-IBC	Plasma	Diagnosis	Up-regulated	Significantly higher in post-menopausal IBC/non-IBC patients	CDKN1B	Proliferation, apoptosis	[37, 78]
miR-19a	IBC	Serum	Prognosis	Up-regulated	High levels of serum miR-19a is a predictive bio-marker for favorable clinical outcome in patients with metastatic HER2+ IBC	Fra-1 and its downstream genes (VEGF, STAT3 and pSTAT3)	Progression, metastasis	[37, 49, 50]
miR-7	IBC	Primary tissue	Prognosis	Up-regulated	High expression was associated with ER (+) status in IBC, also associated with prolonged MFS of IBC patients	EGFR, IGF1R, Wave3	Proliferation, metastasis, invasion	[39, 52]
miR-324-5p	IBC	Primary tissue	Prognosis	Up-regulated	Up-regulation of miR-324-5p was related to prolonged MFS in IBC phenotype	CUEDC2	Invasion, metastasis	[39, 53–55]
miR-21	IBC/non-IBC	Primary tissue	Prognosis	Up-regulated	Increased level of miR-21 was associated with higher grade in IBC, and higher stages and unfavorable molecular subtypes in non-IBC	PDCD4, PTEN, SPRY2 BCL2, TPM1, MASPIN	Migration, invasion, metastasis	[39, 56–59]
miR-205	IBC	Primary tissue	Prognosis	Down-regulated	Down-regulated in IBC and lower expression of miR-205 was associated with shorter distant metastasis-free survival and overall survival	ZEB1, ZEB2, HER3, AMOT, erbB2/erbB3	Proliferation, invasion, metastasis	[60–73]
miR-29a, miR-30b and miR-520a-5p	IBC	Primary tissue	Prognosis	–	Significant associations between specific miRNA target gene expression and patient outcome (DMFS, RFS and OS)	BRWD1, NRIP1, RAPGEF6, TMPO, ABCE1…	Proliferation, invasion, metastasis	[74–76]
miR-520a-5p	IBC	Primary tissue	Prognosis	Up-regulated	Higher levels of IBC specific miR-520a-5p target gene expression with a shorter DMFS, RFS or OS in non-IBC	TMPO, ABCE1, KPNA1, PTP4A2…	Invasion, metastasis	[74]
A 5-miRNA signature (miR-421, miR-486, miR-503, miR-720 and miR-1303)	IBC/non-IBC	Primary tissue	Prognosis	–	This signature is predictive for IBC phenotype with an overall accuracy of 89%, also an independent predictor of poor metastasis-free survival in non-IBC patients	FXR, DPC4/Smad4, ATM, ANK, CCND1, TWIST1, GSK3β, SFRP1, CLDN18	Proliferation, invasion, migration, metastasis	[39, 74, 77, 79–81]
miR-421	IBC/non-IBC	Primary tissue	Prognosis	Up-regulated	Up-regulated in IBC, higher miR-421 expression associated with higher histological grade in non-IBC and IBC and a significant poorer MFS in non-IBC	FXR, DPC4/Smad4, ATM	Proliferation, migration	[39, 82–84]
miR-486	IBC/non-IBC	Primary tissue	Prognosis	Up-regulated	Over-expressed in IBC, higher miR-486 expression levels associated with longer MFS in non-IBC	ANK	Proliferation, apoptosis	[39, 85]
miR-503	IBC	Primary tissue	Prognosis	Up-regulated	Most highly over-expressed in IBC, high miR-503 expression was correlated with higher stage breast cancer	CCND1	Proliferation	[39, 86]
miR-720	IBC	Primary tissue	Prognosis	Up-regulated	Significantly up-regulated in both IBC/nonIBC and IBC/normal	TWIST1	Invasion, migration	[27, 80, 87]
miR-1303	IBC	Primary tissue	Prognosis	Down-regulated	miR-1303 was down-regulated in IBC/non-IBC, and high expression was associated with estrogen receptor positive status in IBC	GSK3β, SFRP1, CLDN18	Proliferation, invasion	[39, 79, 81]

Among these 5 miRNAs, there are 3 miRNAs also relative to some specific characteristics of IBC. According to a few studies before, there are some molecular and clinicopathologic features may influence the level of miRNAs in breast cancer. For example, compared to normal breast tissues, Nassar et al. [38] found that in post-menopausal breast cancer samples miR-155 was up-regulated, which made it a important breast cancer molecular bio-marker for postmenopausal patients. Study conducted by Hamdi et al. [21] also indicated that, when compared with those with nulliparity, miR342-5p was up-regulated in patients who have positive parity history. For non-IBC patients, miR-335 could be an important non-IBC bio-marker for pre-menopausal patients while miR-24 for post-menopausal non-IBC patients, which would contribute to the diagnosis of early onset in non-IBC patients [21].

When it comes to the IBC patients, previous studies have indicated that several miRNAs (such as miR-15a, miR-342-3p and miR-342-5p) were associated with a few molecular and clinicopathologic features (like Her2 status and menopausal state).

### miR-301b

**Fig. 1:**
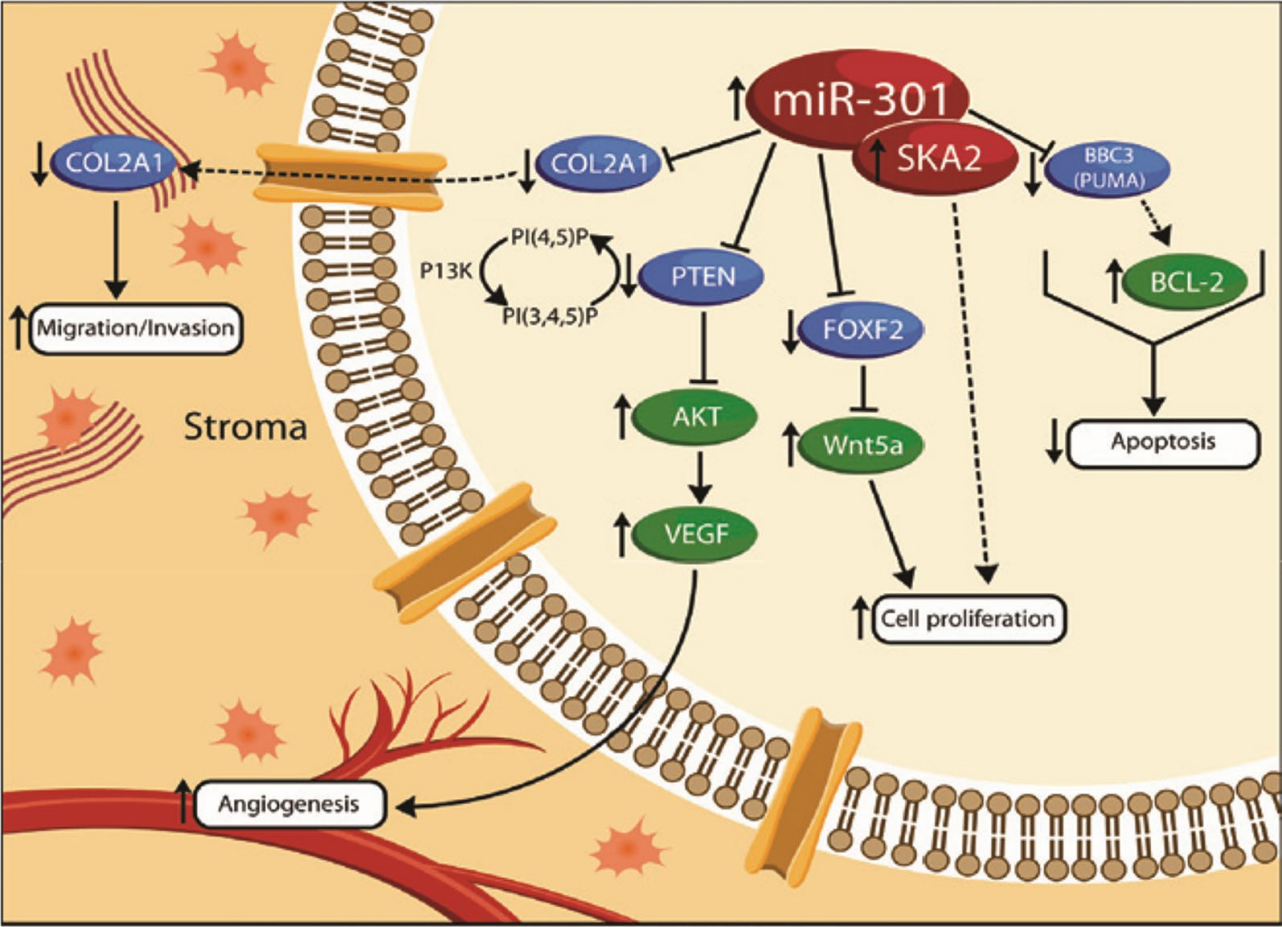
Schema of miR-301 regulated pathways. A proposed model wherein miR-301 overexpression in human breast cancer can downregulate several mRNA targets, including Col2A1, PTEN, FoxF2, and BBC3. In turn, downregulation of Col2A1 can lead to increased migration and invasion. Similarly, PTEN downregulation will lead to Akt activation, which can promote VEGF expression, with increased tumor angiogenesis. Downregulation of FoxF2 can increase expression of Wnt5a, which can promote cell proliferation. In parallel, SKA2 is also co-expressed with miR-301, which can also potentially increase proliferation [40]

Based on the study led by Lerebours et al. [39], miR-301b was observed highly over-expressed in IBC patients when compared to non-IBC. Targeting to FOXF2, BBC3, PTEN, and COL2A1, miR-301b functions in proliferation, invasion, migration and finally local recurrence as well as distant metastasis [40]. Apart from these findings, increased miR-301 expression level was relevant to higher grade in IBC patients, while to ER(−), stage III and higher grade in non-IBC patients [39] (Fig. 1).

### miR-451

miR-451 is found obviously down-regulated in IBC patients when compared to normal sera, providing credible evidence for miR-451 being considered as a new serological bio-marker for IBC, which is derived from a study led by Khouloud et al. and Hamdi et al. [21, 37]. In this regard, one possible explanation accounting for this phenomenon is that exosomes containing miR-451 are cut off 57.5-fold. Consequently, the release of exosomes is lesser extent and the level of miR-451 expression in circulation is lower in IBC patients. This might be either the result of the development progress of tumor or the cause of this lethal disease then leading to the oncogenic of IBC, for which the general mechanism is not clear to date. In addition, according to a few correlation analyses, there is no definite association between miR-451 and other miRNAs. Furthermore, mir-451 showed up an AUC of 0.783 with 80.0% specificity and 81% sensitivity in identifying the IBC patients by an average reduce of 57.5-fold compared to normal sera [21].

**Fig. 2:**
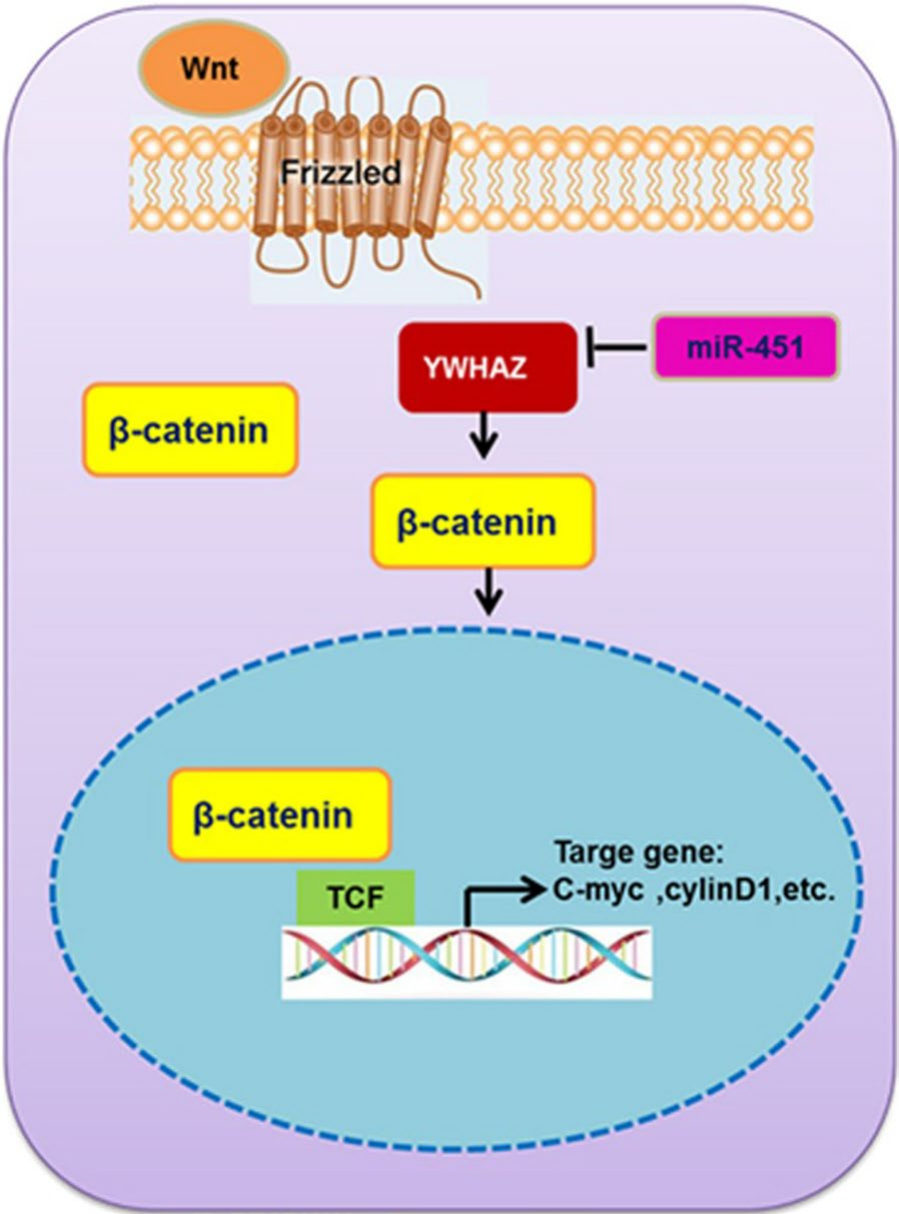
Schema of miR-451 regulated YWHAZ pathway. miR-451 markedly inhibited expression of β-catenin via YWHAZ and subsequently inhibited downstream gene cyclin D1, c-Myc expression, which leads to paclitaxel resistance. Thus, downregulation of miR-451 is able to upregulate YWHAZ expression so that may reduce paclitaxel resistance in IBC [41]

According to a study conducted by Wang et al. [41] YWHAZ was identified as a direct target of miR-451, and downregulation of miR-451 is able to upregulate YWHAZ expression so that may reduce paclitaxel resistance in IBC (Fig. 2). Apart from that, downregulation of miR-451a is also able to upregulate MIF expression and can increase breast cancer cell growth, invasion, and tamoxifen sensitivity, which indicated that the miR-451a/MIF pathway may also be a potential therapeutic target for breast cancer as well as IBC [42].

### miR-15a and HER2 status

**Fig. 3:**
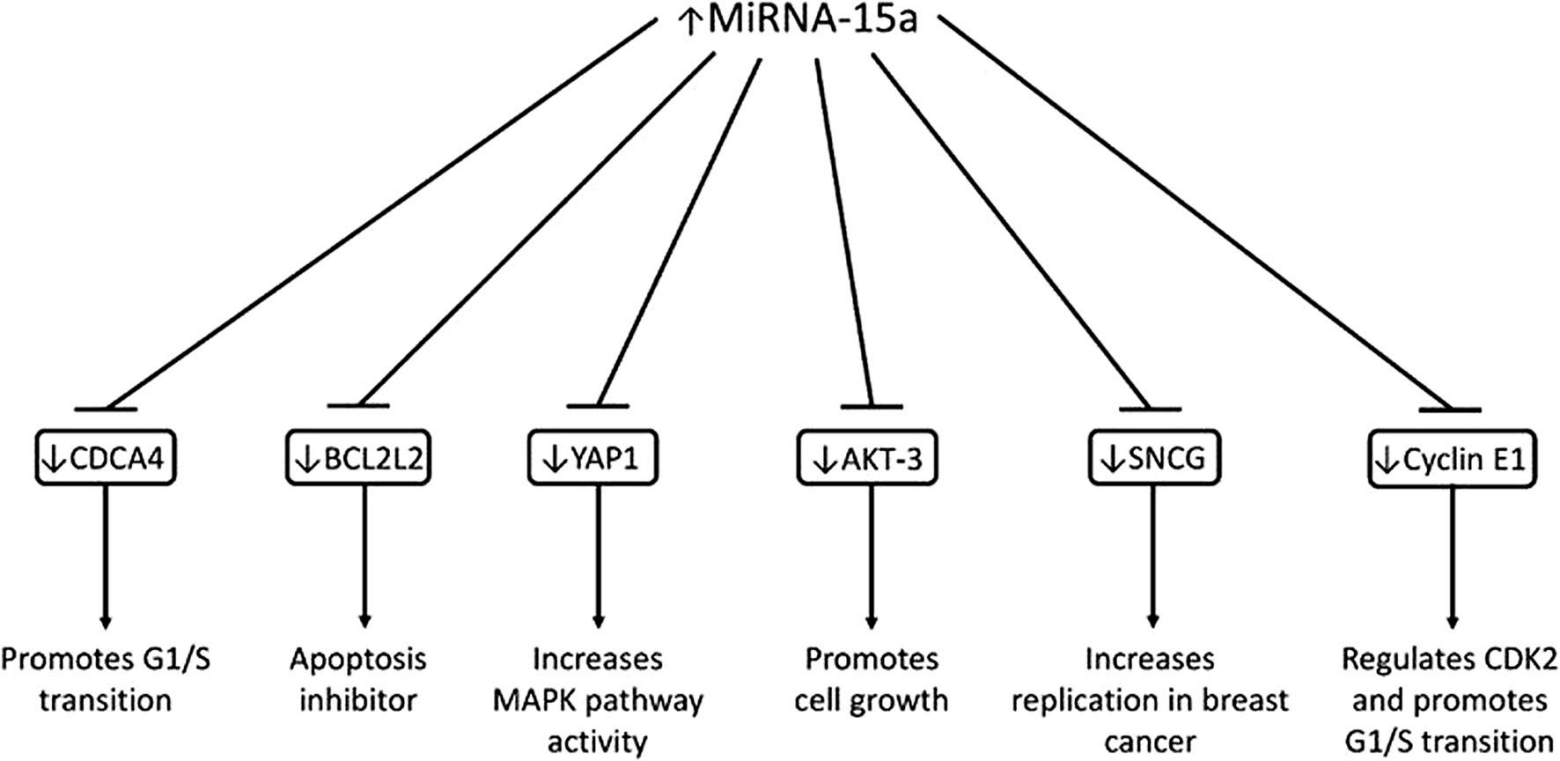
The complete gene targets of miR-15a identified to date and the effects of downregulating these target genes

Levels of miR-15a was positively and closely associated with HER2 status in IBC patients. Targeting to CDCA4, BCL2L2, YAP1, AKT-3 and Cyclin E1, miR-15a has been found to significantly reduce cancer cell survival and aggressiveness (Fig. 3) [43]. According to a few previous studies, in HER2+ IBC patients, serum expression levels of miR-15a were obviously lower than that in HER2− IBC patients, indicating that HER2/neu over-expression in Her2+ IBC patients may change expression levels of miR-15a. Consequently, hypothesis can be concluded that tumor cells that over-expressed HER2 probably make a contribution to the reductive miR-15a expression levels in Her2+ IBC patients’ serum. In view of a research conducted by Cittelly et al. [44] they shown that a clinically momentous oncogenic isoform of HER2 called HER2Δ16 is able to suppress miR-15a. Furthermore, by means of targeting Cyclin E1 directly, miR-15a also play a major role in promoting cell apoptosis and mediating cell-cycle arrest of breast cancer, make it clear that miR-15a possesses tumor suppressive activity in a cell line of breast cancer [45, 46]. In addition, due to the discovery of HER2/neu-induced miR-15a restrain, a better understanding about how HER2/neu over-expression accelerates metastasis and invasion of IBC cells is well provided [37]. All these connections between miR-15a and HER2 status indicate that HER-2 signalization is worthy to have a place in the regulation of miR-15a in IBC and miR-15a can be a considerable molecular bio-marker for assessing HER2 status in IBC [37].

### miR-342-3p, miR-342-5p and menopausal state

Unlike miR-15a, miR-342-3p and miR-342-5p have close relationship with menopausal state of IBC patients. Despite that miR-342-3p and miR-342-5p belong to the identical family, they do not notably correlate in IBC. In pre-menopausal IBC patients, plasma level of miR-342-3p was observed to be lower; while in post-menopausal IBC samples, serum miR-342-5p level was down-regulated. This finding indicating that pre/post-menopausal status may affect miR-342-3p and miR-342-5p expression levels separately. Therefore, the potential of miR-342-3p seems to be more appropriate for pre-menopausal patients while miR-342-5p for post-menopausal IBC patients [37]. Furthermore, Crippa et al. [47] found that miRNA-342 was significantly associated with estrogen receptor (ER) levels, and it is able to regulate BRCA1 expression through modulation of ID4 in breast cancer, which indicates its important role in diagnosis of IBC. However, to validate this hypothesis and to figure out the accurate mechanism of this pathway, more studies should be extended in the future.

## miRNAs predict IBC prognosis

It is widely acknowledged that predictive bio-markers and targeted therapies are still scarce for the prognosis and treatment of IBC, which may be in agreement with the lacking for improvement of survival rates of IBC patients hitherto [21]. Some previous studies have proposed a few characteristics of tumor that have effect on IBC prognosis, such as hormone receptors, histological grade and so on. To predict IBC prognosis more accurately, the prognostic value of miRNA has been much accounted of nowadays.

For breast cancer, a number of published studies have identified a few miRNAs that is of prognostic value. Citing an example of miR-155, Roth et al. [48] found that it is distinguished between healthy people and non-metastatic breast cancer patients through analyzing their serum samples. When it comes to the prognosis of IBC patients, several available miRNAs have also been found (Table 1; Fig. 9).

### miRNAs associated preferable prognosis

#### miR-19a

**Fig. 4:**
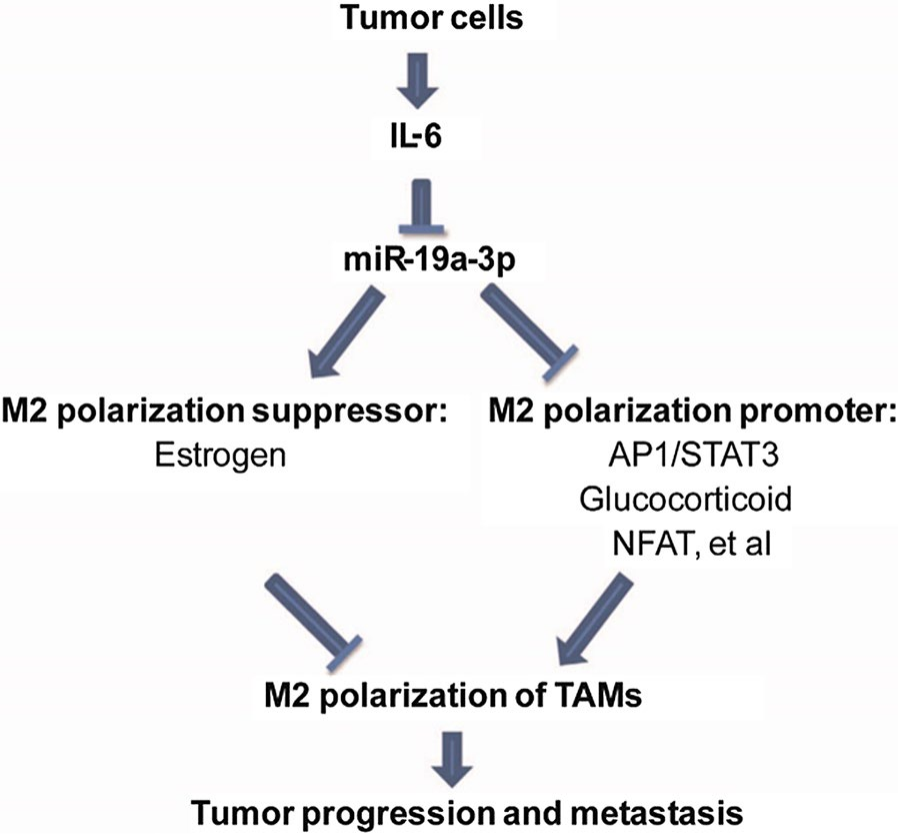
Schema of miR-19a-3p regulated Fra-1 gene pathway. miR-19a-3p, which regulates TAMs in the breast tumor microenvironment, is able to regulate the phenotype of TAMs by targeting the Fra-1 gene and other genes in its downstream signaling pathway. IL-6 activates M2 polarization-related signal pathways of TAMs by inhibiting miR-19a-3p expression. The downregulation of miR-19a-3p expression in TAMs is likely due to TME induction, which promotes transformation of M1 to M2 and results in the enhancement of migration and invasion of breast cancer cells

According to a study conducted by Yang et al. [49] microRNA-19a-3p can inhibit breast cancer progression and metastasis by inducing macrophage polarization through downregulated expression of Fra-1 proto-oncogene (Fig. 4). Apart from that, based on the study led by Anfossi et al. who analyzed plasma samples from IBC, non-IBC and healthy individuals, it was identified that the over-expression of miR-19a in serum was closely associated with a preferable clinical outcome in patients suffered from metastatic HER2+ IBC, which may represent a credible molecular bio-marker for prognosis of IBC patients [37, 50].

#### miR-7

**Fig. 5:**
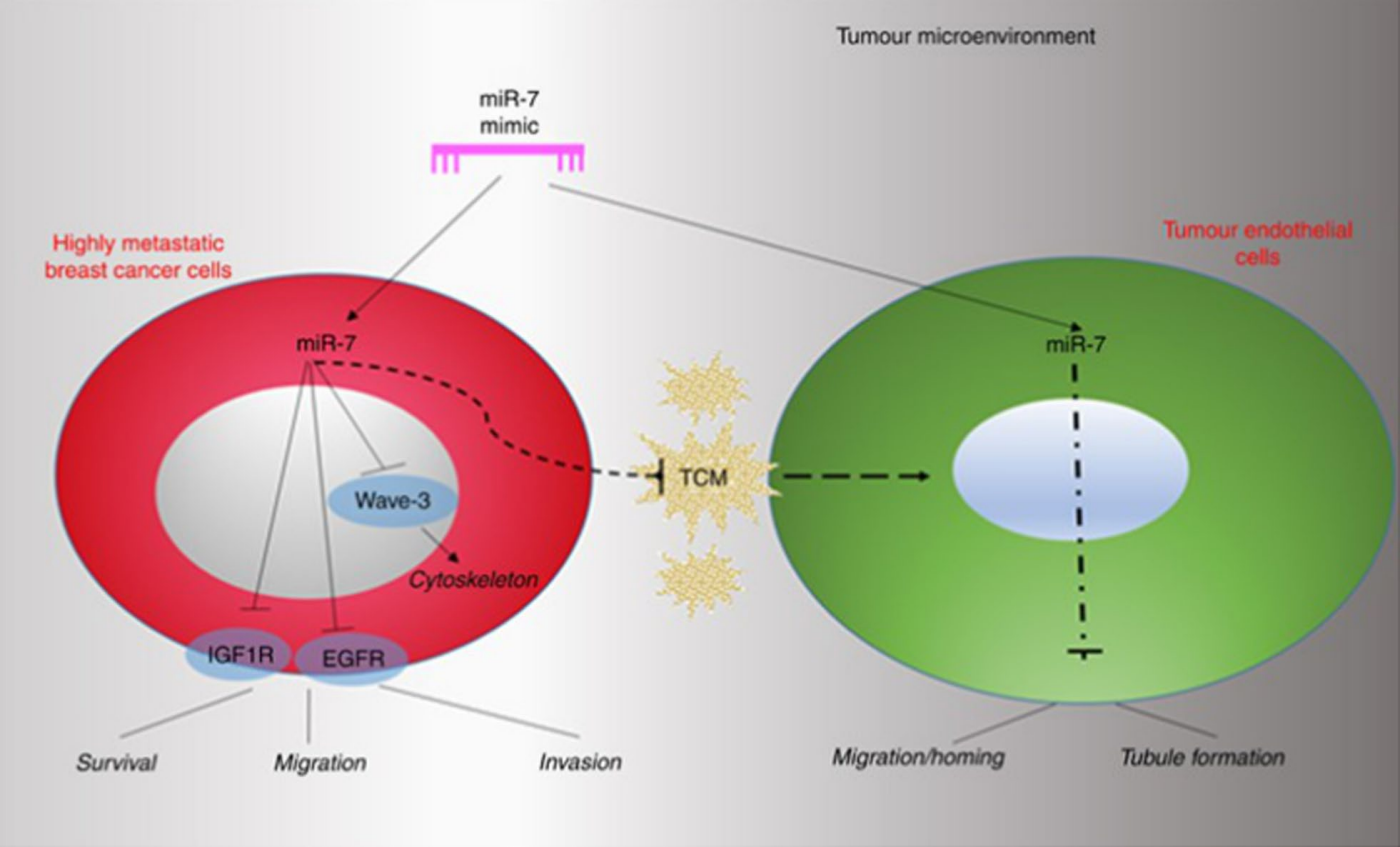
Schema of miR-7 regulated EGFR, IGF1R and Wave3 pathway. miR-7 is expressed at a low level in highly invasive cells, and when added as a form of mimic, it appears to play a more potent role in inhibition of invasive breast cancer cell migration. More importantly, miR-7 mimic indeed has a dual effect as it also significantly inhibits the proliferative, chemotactic and angiogenic-like homing characteristics of endothelial cells especially in response to o chemoattractant factors produced by aggressive breast cancer cells, also suggesting that miR-7 may be developed as an anti-cancer therapeutic potentially capable of suppressing breast cancer metastasis and tumour-associated angiogenesis simultaneously [52]

To a lesser extent, on the strength of its possible tumor-suppressing function, a high level of miR-7 was interrelated to favorable survival and ER(+) status in IBC patients. In addition, miR-7 has been speculated to inhibit the proliferation, migration and invasion of endothelial cells negatively by downregulating the expression of EGFR, IGF1R and Wave3 particularly (Fig. 5), and it is also able to suppress the homing and migration of endothelial cells to more aggressive tumor cell conditions [39, 51, 52].

#### miR-324-5p

Study led by Lerebours et al. [39] showed that up-regulation of miR-324-5p was related to prolonged MFS in IBC phenotype (log rank test: p = 0.0108, HR = 4.37). Although it hasn’t been confirmed that miR-324-5p is associated with cancer samples in human, it was discovered that the oncoprotein HPV16 E5 in cervical epithelial cells could repress the expression of miR-324-5p [53]. A study conducted by Song et al. [54] provided evidence that sinomenine treatment suppressed breast cancer cell invasion and metastasis via regulation of the IL4/miR-324-5p/CUEDC2 axis. Besides, in neuronal tumors, the downregulation of miR-324-5p functioned in inhibiting the oncogenic Hedgehog pathway by influencing carcinogenesis and tumor cell proliferation [55]. However, a large number of experiments are still needed to disclosure its precise mechanism.

### miRNAs associated poor prognosis

#### miR-21

**Fig. 6:**
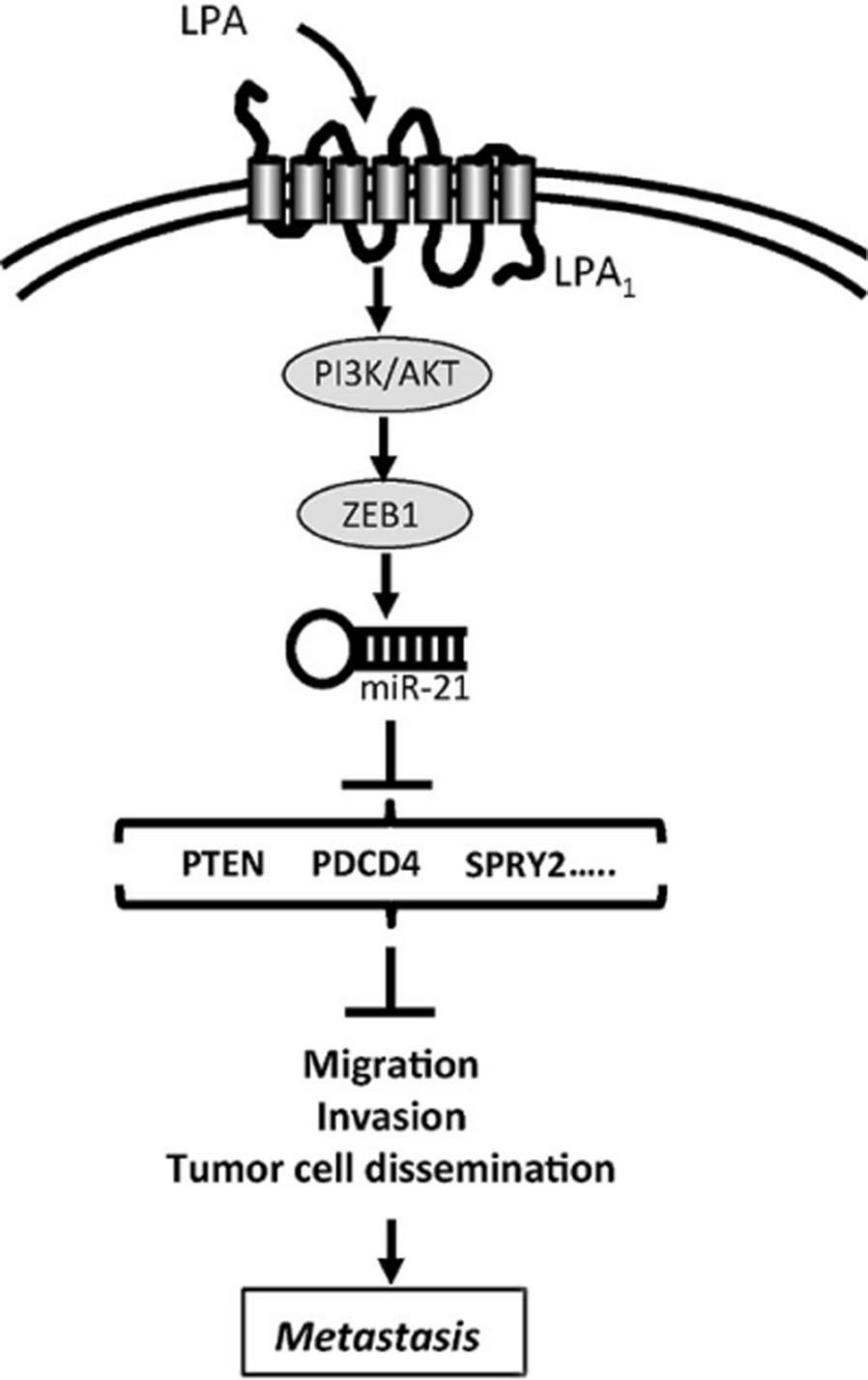
Schema of miR-21 regulated PTEN, PDCD4, SPRY2 pathway. Schematic diagram representing the mechanism of LPA-induced early steps of metastasis formation. Acting on LPA1 receptor LPA activates PI3K/AKT inducing ZEB1 expression and down-stream activation of miR-21 that by inhibiting the expression of anti-metastatic genes (PTEN, PDCD4, SPRY2) induces cell migration, invasion and metastasis dissemination

**Fig. 7:**
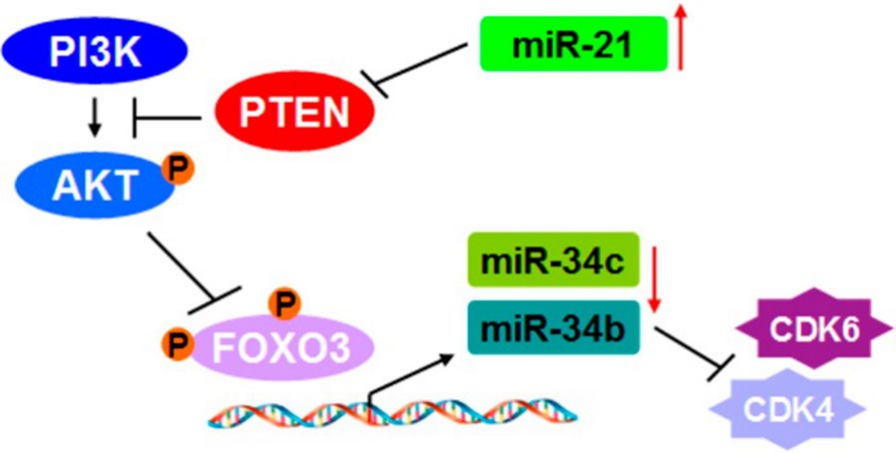
A model of the miR-21-FOXO3a-miR-34b/c signaling in breast cancer. Oncogenic miR-21 up-regulation promoted PI3K/AKT signaling activation through directly inhibiting PTEN expression, a suppressor of PI3 K/AKT. The activation of AKT phosphorylates FOXO3a resulting in relocalization of FOXO3a proteins from nucleus to the cytoplasm. Nuclear FOXO3a down-regulation reduced the binding efficiency of FOXO3a in the promoter of miR-34b/c leading to decreases in the expression levels of miR-34b and miR-34c in cells

As an oncogenic miRNA, miR-21 has been confirmed to be recurrently involved in occurrence and development of tumor. This process is achieved mainly by targeting in specific tumor-suppressor genes such as PDCD4, PTEN, SPRY2 (Fig. 6) [56, 57]. Liu et al. [58] also found miR-21-FOXO3a-miR-34b/c signaling pathway in breast cancer (Fig. 7). Consequently, an increased level of miR-21 is related to poor DFS (disease-free survival) in breast cancer [59], higher grade in IBC patients, as well as undesirable molecular subtypes and higher stages in non-IBC patients [39].

#### miR-205

The expression level of miR-205 was reported to variate from different tumors. Among the samples of bladder cancer [60], endometrioid adenocarcinoma [61] and lung cancer [62], miR-205 was observed to be over-expressed. While in human samples of breast cancer [63–65], melanoma [66] and prostate cancer [67], it was down-regulated.

**Fig. 8:**
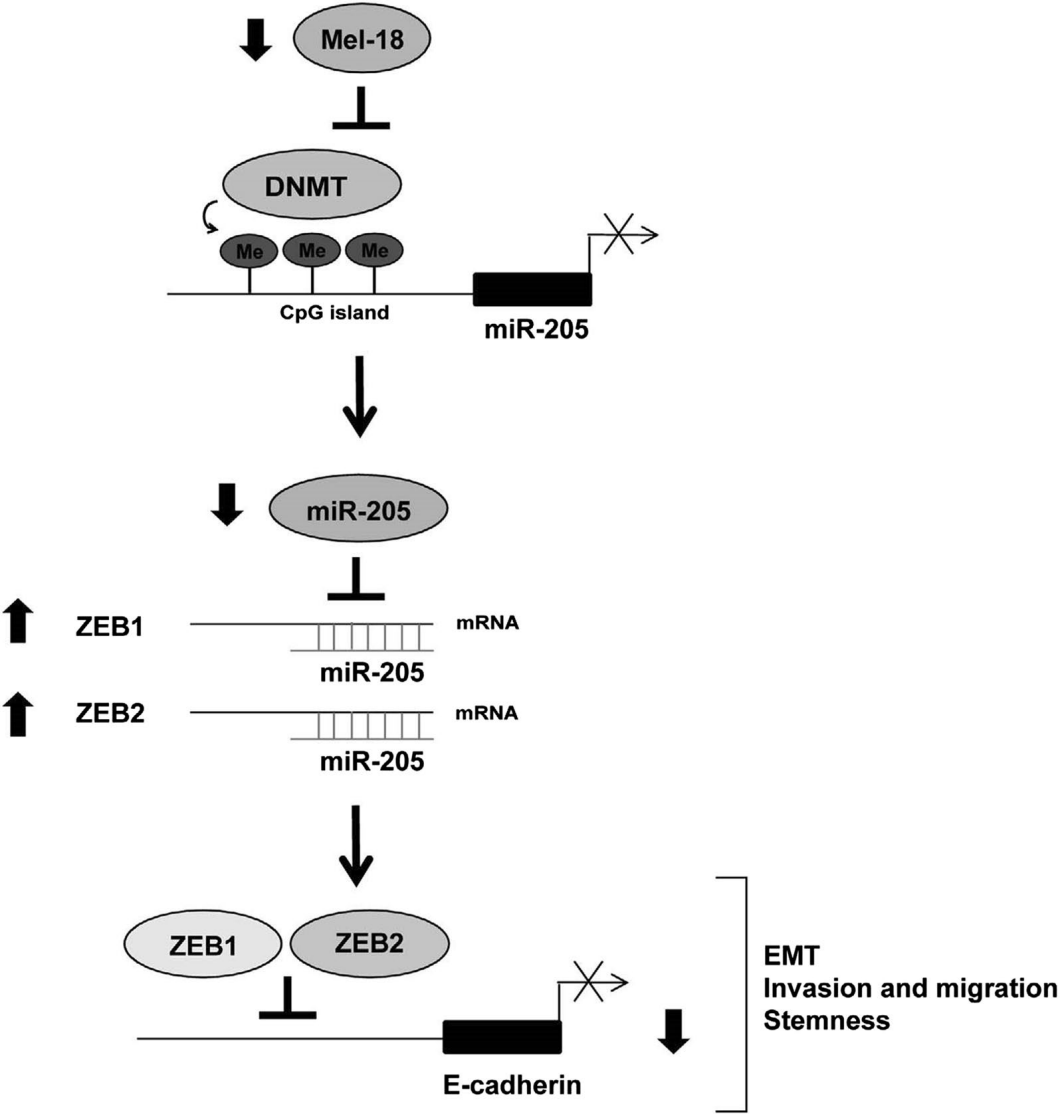
Schema of miR-205 regulated ZEB1, ZEB2, EMT pathway. A model for the regulatory mechanism of EMT by Mel-18. Loss of Mel-18 in breast cancer leads to hypermethylation of the CGrich region of the miR-205 promoter by regulating recruitment of DNMT family proteins to the miR-205 proximal promoter, and results in downregulation of miR-205 expression. This epigenetic repression of miR-205 leads to upregulation of the target genes, ZEB1 and ZEB2, and consequently activates ZEB1- and ZEB2-mediated repression of E-cadherin transcription. Thus, loss of E-cadherin by the miR-205/ZEB1-ZEB2 cascade following Mel-18 depletion in breast cancer induces the EMT and tumor invasion

A research of 47 individuals (23 IBC and 24 non-IBC human samples) conducted by Huo et al. [68] suggested that the low expression of miR-205 was associated with advanced breast cancer, particularly in inflammatory breast cancer. By studying cultured breast cancer cell-lines, miR-205 is capable to inhibit proliferation, invasiveness, anchorage-independent growth and clonogenic survival, which is in agreement with its functions in the process of tumor growth, invasion, and metastasis [63]. Based on studies published before, this process is achieved mainly by targeting ZEB1, ZEB2, EMT (Fig. 8) [69], HER3, AMOT, erbB2/erbB3 and so on [60–73].

Recent published studies have found that in mouse models, yield of miR-205 through nanoliposomes is able to make breast cancer cells more sensitive to radiation therapy [70]. Apart from that, an antioxidant-induced expression of miR-205 can modulate epithelial–mesenchymal transition negatively, which is an significant early-step in tumor cell metastasis [71]. Therefore, A lower expression level of miR-205 was related to worse DMFS (distant metastasis-free survival) and OS (overall survival), which indicating that miR-205 can serve as a promising prognostic bio-marker as well as a possible therapeutic target for inflammatory breast cancer.

#### The IBC-specific miRNAs (miR-29a, miR-30b and miR-520a-5p)

Based on the first-published integrated study of mRNA and miRNA expression in IBC, Auwera et al. [74] discriminated a set of 13 miRNAs, whose expression is analyzed to be distinguished between IBC and non-IBC patients. Some of them were associated with a dismal disease prognosis, making these miRNAs promising molecular predict bio-markers for the IBC subtype.

When compared with non-IBC, scholars found that over-expression of 6 miRNAs (miR-335, miR-337-5p, miR-451, miR-486-3p, miR-520a-5p and miR-548d-5p) was identified in the IBC patients, while 7 miRNAs (miR-15a, miR-24, miR-29a, miR-30b, miR-320, miR-342-5p and miR-432-3p) were obviously down-regulated. Among these 13 miRNAs, 3 miRNAs (miR-29a, miR-30b and miR-520a-5p) related genes were able to serve as predicted targets. According to a gene set enrichment analysis, their important enrichment in various biological processes was discriminated, which was associated closely to signal transduction and cell proliferation.

For miR-29a, its target genes function in DNA methyltransferase activity mainly, which is demonstrated in lung cancer [75] and acute myeloid leukemia [76].

For miR-30b, its target genes were observed to be related to insulin receptor signaling, whereas target genes of miR-520a-5p proved to be interrelated to cell proliferation. The target genes of 3 miRNAs mentioned above such as BRWD1, NRIP1, RAPGEF6, TMPO, ABCE1 have close ties to patient outcome (OS, RFS and DMFS). Particularly, higher target genes levels of miR-520a-5p relevant to shorter OS, RFS and DMFS, demonstrating a significantly promising role of miR-520a-5p as a prognostic bio-marker in IBC [74].

#### 5-miRNAs signature (miR-421, miR-486, miR-503, miR-720 and miR-1303)

A minimal group of 5-miRNAs is composed of miR-421, miR-486, miR-503, miR-720 and miR-1303, which is highly predictive for IBC, exerting a tremendously remarkable discriminating value on classification performance (accuracy of 89%, p < 0.0001) [39]. Unlike the common breast cancer biological parameters such as molecular subtype and grade, the 5-miRNAs expression signature was able to distinguish the cancer according to their potential risks for recurrence and aggressiveness, serving as a metastasis sign on the basis of tumor spreading potential. According to a classification based on 5-miRNA signature led by Lerebours et al. [39] tumors of IBC-like group displayed clinic-pathological characteristics related to tumor aggressive behavior such as negative hormone receptors, higher histological grade and distant metastases. Furthermore, when compared with MFS between these two groups, survival analyses manifested that tumors of IBC-like group were obviously related to poor prognosis (log rank test: p50.0005). Therefore, this 5-miRNAs signature can be regarded as a promising and predictive bio-marker of IBC phenotype.

Furthermore, according to a multivariate-analysis which studied all momentous prognostic factors of non-IBC, the 5-miRNAs signature was an independent predictor of unfavorable metastasis free survival [39]. Thus, a specific gene signature resulted from these 5-miRNAs can be a predictive bio-marker for IBC, also responsible for breast cancer aggressiveness generally [74, 77].

This hypothesis was tested by Lerebours et al. [39] in the sample of 95 non-IBC patients. In this study, this signature distinguished patients into two groups. Between the two groups, the molecular subtype and grade of each group were equivalent whereas the outcome and stage were obviously different. Despite mis-classified samples, tumors of IBC-like group revealed aggressive clinicopathological characteristic with negative hormone receptors, high histological grade and distant metastases. By comparing MFS between the two groups, Lerebours et al. [39] discovered that IBC-like group had close ties to worse prognosis. Therefore, this 5-miRNA signature can consequently serve as predictive bio-marker of IBC phenotype.

## Conclusions

**Fig. 9:**
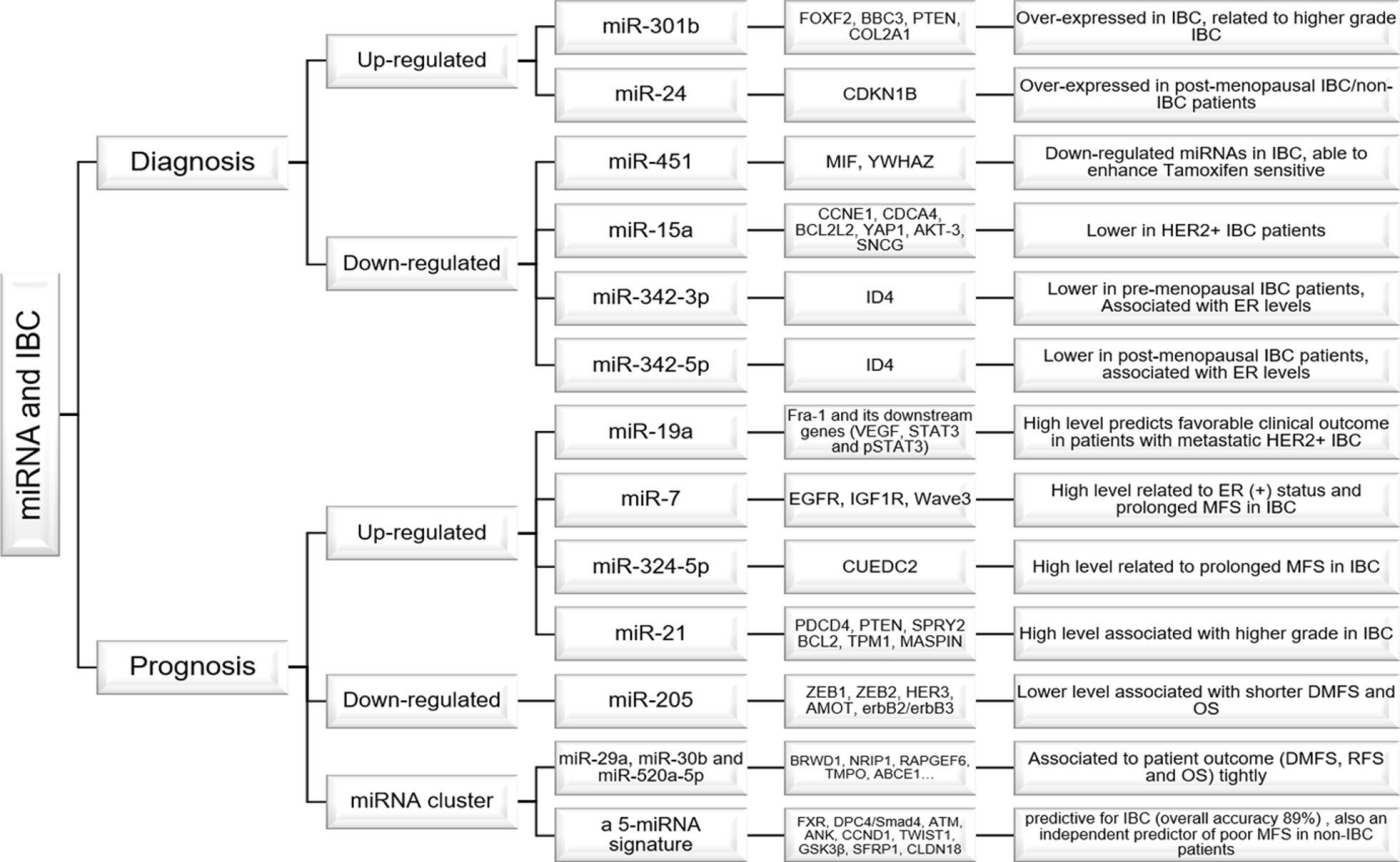
Schema of miRNA and IBC

In summary, if verified in future, further studies, miR-15a, miR-342-3p, miR-342-5p and miR-15a were associated with a few clinicopathologic features (such as menopausal state and Her2 status); miR-451, miR-301b, miR-503, miR-720 and miR-486 could be considered and used as bio-markers for diagnosis of IBC; miR-19a, miR-21, miR-7, miR-205, miR-29a, miR-30b, miR-520a-5p and a 5-miRNA signature (miR-421, miR-486, miR-503, miR-720 and miR-1303) may serve as molecular bio-markers for prognosis of IBC. These findings may provide a better understanding of the miRNAs signatures and roles in IBC, identifying several novel and minimally invasive bio-markers for diagnostic and prognostic strategies by the means of targeting specific miRNAs (Fig. 9).

However, there are still many limitations in this area. Firstly, most relevant literature is currently limited to studies on single or several miRNAs and there is a lack of a comprehensive network. Secondly, most of the previous studies on miRNAs are confined to detecting differences of miRNAs expression levels between IBC and non-IBC samples, thus a specific IBC signature and the mechanism have not been completely illuminated yet. More importantly, the detection methods and the sample source of miRNAs are different between these studies, making the comparability of the results is not strong enough. We still have a long way to go to translate these laboratory findings to the clinic, making miRNAs practicable bio-markers for IBC patients.

Therefore, future larger studies are keenly awaited and required to verify these preliminary findings and further permit us to figure out whether and how miRNAs alterations are significantly associated to risk, onset and progression of the inflammatory breast cancer, and identify their potential as promising molecular bio-markers for early IBC diagnosis, prognostic and accelerate the progress of novel strategies for IBC therapy and prevention.

## Abbreviations

AJCC: American joint committee on cancer
IBC: inflammatory breast carcinoma
miR/miRNA: microRNA
LABC: locally advanced breast cancer
LASP1: gene encoding LIM and SH3 domain protein 1
HER2: human epidermal growth factor receptor 2
EGFR: epidermal growth factor receptor
MFS: metastasis-free survival
DFS: disease-free survival
DMFS: distant metastasis-free survival
OS: overall survival
PDCD4: gene encoding protein programmed cell death protein 4
BCL2: gene encoding B cell lymphoma 2
TPM1: gene encoding tropomyosin alpha-1 chain
MASPIN: gene encoding mammary serine proteinase inhibitor
PTEN: gene encoding phosphatase and tensin homolog
FOXF2: gene encoding forkhead box F2
BBC3: BCL2, gene encoding binding component 3
COL2A1: gene encoding collagen type II alpha 1 chain
MIF: gene encoding macrophage migration inhibitory factor
YWHAZ: gene encoding tyrosine 3-monooxygenase/tryptophan 5-monooxygenase activation protein zeta
CCNE1: gene encoding cyclin E1
CDCA4: gene encoding cell division cycle associated 4
BCL2L2: gene encoding BCL2 Like 2
YAP1: gene encoding yes associated protein 1
AKT-3: gene encoding AKT serine/threonine kinase 3
SNCG: synuclein gamma
ID4: gene encoding inhibitor of DNA binding 4, HLH protein
CDKN1B: gene encoding cyclin-dependent kinase inhibitor 1B (P27, Kip1)
Fra-1: a pro-oncogene whose downstream genes are VEGF, STAT3 and pSTAT3
TAMs: tumor-associated macrophages
IGF1R: gene encoding insulin like growth factor 1 receptor
Wave3: gene encoding WAS protein family member 3
CUEDC2: gene encoding CUE domain containing 2
SPRY2: gene encoding sprouty RTK signaling antagonist 2
ZEB1/ZEB2: gene encoding zinc finger E-box binding homeobox 1/2
AMOT: gene encoding angiomotin
erbB2/erbB3(HER3): Erb-B2 receptor tyrosine kinase 2/3
BRWD1: bromodomain and WD repeat domain containing 1
NRIP1: gene encoding nuclear receptor interacting protein 1
RAPGEF6: gene encoding rap guanine nucleotide exchange factor 6
TMPO: gene encoding thymopoietin
ABCE1: gene encoding ATP binding cassette subfamily E member 1
KPNA1: gene encoding karyopherin subunit alpha 1
PTP4A2: gene encoding protein tyrosine phosphatase type IVA, member 2
ATM: gene encoding ATM serine/threonine kinase
ANK: gene encoding ankyrin
CCND1: gene encoding cyclin D1
TWIST1: twist family BHLH transcription factor 1
GSK3β: gene encoding glycogen synthase kinase 3 beta
SFRP1: gene encoding secreted frizzled related protein 1
CLDN18: gene encoding claudin 18
